# Spooky Action at a Temporal Distance

**DOI:** 10.3390/e20010041

**Published:** 2018-01-10

**Authors:** Emily Adlam

**Affiliations:** Basic Research Community for Physics, Leipzig 04315, Germany; eadlam90@gmail.com

**Keywords:** quantum foundations, nonlocality, retrocausality, Bell’s theorem

## Abstract

Since the discovery of Bell’s theorem, the physics community has come to take seriously the possibility that the universe might contain physical processes which are spatially nonlocal, but there has been no such revolution with regard to the possibility of *temporally* nonlocal processes. In this article, we argue that the assumption of temporal locality is actively limiting progress in the field of quantum foundations. We investigate the origins of the assumption, arguing that it has arisen for historical and pragmatic reasons rather than good scientific ones, then explain why temporal locality is in tension with relativity and review some recent results which cast doubt on its validity.

## 1. Introduction

Since the discovery of Bell’s theorem [1], the physics community has broadly come to take seriously the possibility that the universe might contain physical processes which are spatially nonlocal. However, there has been no such revolution with regard to “temporal locality”, i.e., the assumption that the probabilities attached to the outcomes of a measurement performed at a given time depend only on the state of the world at that time. Indeed, temporal locality remains almost ubiquitous in the way that scientists think about science and about what constitutes a reasonable scientific hypothesis.

An assumption so widespread and yet so infrequently justified is in serious danger of becoming a dogma. While it is true that temporal locality has previously been recognised as problematic by parts of the physics community, we argue that this recognition is not sufficiently widespread and that the assumption is actively limiting progress in the field of quantum foundations. In this article, we investigate the origins of this way of thinking about physics, arguing that it has become dominant for historical and pragmatic reasons rather than good scientific ones. We then explain why temporal locality is in tension with relativity, and review some recent results which cast doubt on the status of temporal locality in modern physics.

## 2. Temporal Locality

### 2.1. Definition

In seeking to set out a definition of temporal locality, a natural starting point is the standard mathematical definition of spatial locality [2,3]:

**Definition** **1.** ***Spatial Locality:*** *Suppose that two observers, Alice and Bob, perform measurements on a shared physical system: Alice performs a measurement with setting a and obtains a measurement outcome A, while Bob performs a measurement with measurement setting b and obtains a measurement outcome B. Let λ be the joint state of the shared system prior to the two measurements. Then:*

p(A,B|a,b,λ)=p(A|a,λ)p(A|b,λ)

We can straightforwardly apply this language to the temporal case:

**Definition** **2.** ***Temporal Locality:*** *Suppose that two observers, Alice and Bob, perform measurements on a shared physical system. At some time $t_{a}$, Alice performs a measurement with measurement setting a and at some time $t_{a}+\delta$ she obtains a measurement outcome A; likewise, at some time $t_{b}$, Bob performs a measurement with measurement setting b and at some time $t_{b}+\delta$ he obtains a measurement outcome B. Let $\lambda (t_{a})$ be the state of the world at time $t_{a}$ and let $\lambda (t_{b})$ be the state of the world at time $t_{b}$. Then:*

$$p\left(A,B|a,b,\lambda \left(t_{a}\right),\lambda \left(t_{b}\right)\right)=p\left(A|a,\lambda \left(t_{a}\right)\right)p\left(A|b,\lambda \left(t_{b}\right)\right)$$

The central idea of this definition is that in a temporally local world there would be “no action at a temporal distance”, i.e., all influences on a measurement outcome would be mediated by the state of the world immediately prior to the measurement. Of course, the definition does not lead to any specific theoretical constraints without some specification of what is included in “the state of the world at time *t*”, but in this article we will not single out any unique way of characterising this state: instead, we will set out a range of options, acknowledging that there are a number of related concepts floating around in modern physics which might reasonably be subsumed under the heading of temporal locality.

It is helpful to approach this range of possibilities by describing some different ways in which physics might *fail* to be temporally nonlocal. First, a theory might fail to be temporally local by postulating non-Markovian laws, meaning that the results of a measurement at a given time can depend on facts about earlier times even if there is no record of those facts in the state of the world immediately prior to the measurement. Note that this is possible only within a theory in which the state of the world at time *t*, if such a thing exists, does not always contain complete information about everything that has happened before *t*. Alternatively, a theory might fail to be temporally local by being retrocausal, meaning that the results of measurements at a given time may depend in part on information about the future. We reinforce that retrocausality does not immediately imply temporal nonlocality: a retrocausal theory is temporally nonlocal only if it tells us that the result of a measurement can depend on facts about the future even if there is no record of those facts in the state of the world immediately prior to the measurement. Therefore this type of temporal nonlocality is possible only within a theory in which the state of the world at time *t*, if such a thing exists, does not always contain complete information about everything that happens after *t*—in particular, it must not be the case that the state of the world immediately prior to the measurement already contains a record of the future outcome of the measurement, as for example in theories which are deterministic in the traditional sense, meaning that the state of the world at a given time determines everything that happens at later times. Finally, a theory might fail to be temporally local by being atemporal, meaning that the course of history is determined “all at once” by external, global laws of nature, in much the same way as the rules of the game of sudoku apply to the whole grid at once rather than dictating the entries column by column from left to right. In such a theory, the result of a measurement at a given time may depend on global facts even if there is no record of those facts in the state of the world immediately prior to the measurement, and thus an atemporal theory will usually be temporally nonlocal, unless of course the theory tells us that the state of the world at time *t* always contains complete information about the history of the entire universe. Each of these alternatives singles out a different sense of temporal (non-)locality, and all three raise interesting possibilities for new ways of thinking about physics.

### 2.2. Motivation

Although physicists are certainly aware that the assumption of temporal locality is problematic, as a methodological principle it remains very widespread in the field. Although, presumably, some physicists would fight to the death for temporal locality, it seems likely that many others retain it simply because they regard it as a harmless simplification. However, we argue that the assumption is by no means harmless: temporal locality is deeply woven into many of the key results on which our present understanding of the interpretation of quantum theory is founded, and unpicking it would require a radical reinterpretation of the significance of those results.

In particular, much recent work in quantum foundations has been based within the “ontological models” framework introduced by Spekkens in [4], where it is supposed that every system has a single real “ontic state”, which determines the probabilities for the outcomes of any measurement on that system. An ontological model thus consists of a space Λ of ontic states λ, a set of probability distributions $\mu^{P}\left(\lambda \right)$ giving the probability that the system ends up in the state λ when we perform the preparation procedure *P*, a set of response functions ${\vec{\xi }}_{M,O}\left(\lambda \right)$ giving the probability that we obtain outcome *O* when we perform measurement *M* on a system whose ontic state is λ, and a set of column-stochastic matrices $T^{X}$ representing the way in which the ontic state is transformed when some operation *X* is applied to the system. Note that talk of “ontic states” does not imply that we are postulating the existence of hidden variables, because the “ontic state” could simply be the quantum state [5]. It should also be reinforced that one can make use of the formalism of ontological models without necessarily interpreting it as an attempt at a faithful representation of reality—Spekkens himself prefers to regard it as a classification schema which enables us to give precise mathematical definitions for concepts like contextuality [6]. Nonetheless, it seems to be the case that this formalism, or something close to it, *is* often regarded as a description of reality, and indeed as the only possible way of describing reality—for example, in [7], it is claimed that any model in which correlations are not explained by appeal to ontic states should not really be regarded as a realist model at all.

The ubiquity of this method of analysis matters, because the ontological models framework is explicitly temporally local. Not only that, temporal locality is the founding principle of the approach: the entire project of constructing an ontological model is premised on the assumption that measurement results must depend only on the information available in the ontic state at the time of the measurement. Consequently, temporal locality is the keystone of a number of influential results parsed in the language of ontological models, such as Spekkens’ generalized proofs of contextuality [4], the Colbeck–Renner theorem [8], Hardy’s theorem [9], and the Pusey–Barrett–Rudolph (PBR) theorem [10].

As a case study, let us consider the PBR theorem, which states that no model in which the quantum state is not an “element of reality” can reproduce all the quantitative predictions of quantum mechanics. Now, the term “element of reality” is a reference to a definition set out by Harrigan and Spekkens [11], but although this definition refers only to instantaneous facts, the proof of the PBR theorem depends implicitly on assumptions not only about states at a given time, but about the persistence of those states over time: PBR write that if there exists a set of four preparation procedures which all have some probability of preparing the same ontic state, then when this state is prepared, “*the measuring device is uncertain which of the four possible preparation methods was used, and on these occasions it runs the risk of giving an outcome that quantum theory predicts should occur with probability 0*” [10]. This makes it clear that the argument also requires the assumption that the outcome of the measurement can depend on facts about the system’s preparation only via the mediation of an intervening state, so the PBR theorem should really be glossed as follows: *either* the quantum state is ontological, *or* some quantum measurement results must depend in a temporally nonlocal way on events at other times. In this context, then, the assumption of temporal locality is decidedly non-trivial—for example, anyone who wishes to push back against the ontological picture of quantum states should certainly be raising questions about this assumption.

Moreoever, most mainstream interpretations of quantum mechanics, including the Everett interpretation, spontaneous collapse models and the de Broglie Bohm approach, are prima facie temporally local. (We do not mean to suggest that these models could not be phrased in a temporally nonlocal way, nor even to assert that this has not already been done somewhere in the literature, but it does seem to be the case that temporal nonlocality is not a central feature of any of these interpretations). This suggests that fully embracing temporal nonlocality might open up untapped possibilities for the interpretation of quantum theory, and hence the whole landscape of quantum foundations becomes markedly different when temporal nonlocality is taken seriously.

## 3. Origins

Given that temporal locality plays such a key role in our modern understanding of quantum theory, it is important to understand the intellectual history of this idea. In this section, we argue that a number of historical and psychological factors are likely to have contributed to its prominence; indeed, temporal locality is, in a sense, built into the very structure of physics. Consider the long tradition of presenting theories in terms of their kinematics (the space of physical states postulated by the theory) and their dynamics (the set of laws by which these states evolve, according to the theory). This distinction can be traced back at least to Newton, who may have been the first to make a clear distinction between laws and initial conditions [12], and since Newton’s time the formulation has become widespread: it is now almost mandatory to introduce a new physical theory by setting out a space of physical states and a set of evolutionary laws [4]. However, a physical state is, almost by definition, that which carries information from one time to another by means of its dynamical evolution, and thus by employing this mode of presentation we are already very close to assuming that information about one time can influence the results of measurements at other times only via a mediating physical state, thus ruling out temporal nonlocality almost by fiat. Temporal locality is thus very deeply ingrained in the way physicists are taught to think about physics.

There are also straightforward pragmatic reasons why temporal locality should have gained such ascendancy in science. After all, we ourselves are local agents—if we wish to influence events at a spatial or temporal distance we must do so via some spatiotemporally continuous process of mediation—and the fact that these constraints are, for us, so immediate and insurmountable naturally leads us to imagine that the laws of nature must be subject to similar constraints. The empirical results of quantum mechanics, such as the violation of Bell’s inequality, have give us convincing reasons to question the resulting attachment to *spatial* locality, but *temporal* nonlocality has not thus far been subject to the same level of analysis and hence lives on in the ways we think and talk about quantum mechanics. Furthermore, as scientists, our primary practical interest is in formulating laws which enable us to predict the future given our knowledge of the present state of the world, and it is easy to move from the fact that most of the laws proposed by physicists have this form to the conclusion that the true underlying laws of nature must take the same form. However, it would be naive to suppose that the true laws of nature look exactly like the type of laws that human agents are most interested in formulating: as Wharton puts it: “There’s one last anthropocentric attitude that needs to go, the idea that the computations we perform are the same computations performed by the universe”. Assuming that our point of view is not central to the universe, it would be highly suspicious if the laws of nature were to be arranged so conveniently for us.

It also seems likely that certain elements of temporal locality have their origin in the viewpoint known in academic philosophy as “presentism”, which holds that the only things which are real are the things which exist now [13,14,15]. A realist about science will clearly want to insist that measurement results can depend only on things that are real, and hence a realist who subscribes to presentism is compelled to believe that measurement results can depend only on facts about the world immediately prior to the measurement. Presentism is a very old philosophical idea, appearing in the writings of Aristotle and St. Augustine, and playing an important role in Buddhist philosphy, although with the advent of special relativity it has gone somewhat out of vogue as an explicit philosophical thesis: much has been written on the question of whether or not relativity makes presentism untenable [16,17,18,19], but whether or not the two can be formally reconciled, they are certainly in tension with one another. Nonetheless, although there are few modern physicists who would self-describe as presentists, the intuitive picture of the present as somehow specially privileged remains hard to shake, and it is likely that some element of this way of thinking contributes to the general conviction that scientific theories should respect temporal locality.

We reinforce that although these historical and psychological observations go some way towards explaining why our scientific theories tend to be temporally local, they do not offer any epistemic justification for thinking that the world actually *is* temporally local. Of course, it may be the case that some epistemic justification can be provided, but if such a justification exists it is certainly not commonly known and hence cannot be regarded as the main reason why our theories exhibit this feature. This indicates that the prominence of temporal locality in our standard approaches to physics may not be entirely rational and perhaps deserves greater scrutiny than it has thus far received.

### The Pragmatic Argument

At this juncture, a defender of temporal locality might wish to suggest a different type of justification, using pragmatic rather than epistemic arguments. In particular, one might worry that if we accept that events at this moment may depend on events at any point in the past or future, it will become very difficult to track all the variables which might be relevant to the outcome of an experiment, and the whole scientific enterprise will be under threat. Indeed, similar objections were raised by Einstein concerning *spatial* nonlocality [20]:

An essential aspect of this arrangement of things in physics is that they lay claim, at a certain time, to an existence independent of one another, provided these objects “are situated in different parts of space”. Unless one makes this kind of assumption about the independence of the existence (the “being-thus”) of objects which are far apart from one another in space ... physical thinking in the familiar sense would not be possible. It is also hard to see any way of formulating and testing the laws of physics unless one makes a clear distinction of this kind.

However, despite Einstein’s concerns, it has not proven to be impossible to formulate a theory which allows for spatial nonlocality, because the nonlocal relations between events are governed by laws which enable us to identify regularities in patterns of dependence even between spatially separated events. Likewise, in principle it would not be impossible to move forward with a theory which allows for temporal nonlocality, provided that events at a time depend on events at other times in some regular, formalisable way—indeed, we already have a way of tracking patterns of dependence both temporally and spatially, since the quantum state gives a concise summary of all the information about the history of a system which we know to be relevant to the results of future measurements performed on that system. Therefore the assumption of temporal locality is not forced upon us by practical considerations, and it behoves us to consider the possibility that an explicitly temporally nonlocal theory might enable us to identify and track further regularities.

## 4. Relativity

In addition to these general concerns about epistemic rationality, there are also more specific technical reasons to be sceptical about temporal locality. In particular, as we describe in this section, temporal locality is in tension with both special and general relativity.

### 4.1. Special Relativity

The astute reader will already have noticed a problem with our definition of temporal locality: special relativity tells us there is no unique, observer-independent fact about what constitutes the state of the world at a given time [21], and hence the category “the state of the world at time *t*” is not even well-defined. It is possible to dodge this problem if we are working with a theory which is also *spatially* local, since the probabilities for the outcome at time t+δt of a measurement which begins at a time *t* will then depend only on the state of the world at a fixed spacetime point, i.e., the spatiotemporal location at which the measurement begins, which is well-defined even in a relativistic context. However, the combination of spatial nonlocality, temporal locality and special relativity is straightforwardly inconsistent, since an instance of spatial nonlocality becomes an instance of temporal nonlocality under a change of reference frame.

This fact has consequences for many approaches to the interpretation of quantum mechanics. It is the main stumbling block for the de Broglie–Bohm pilot wave interpretation of quantum mechanics, which combines spatial nonlocality with temporal locality and consequently fails to be relativistically covariant in its standard form [22]. Similarly, Tumulka recently put forward what was intended to be a relativistically covariant version of the Ghirardi–Rimini–Weber (GRW) spontaneous collapse model [23], based on Bell’s GRW flash ontology in which the point-like collapse events rather than the quantum states are regarded as fundamental [24], but it was subsequently pointed out by Gisin and Esfield that it is not possible to give a Lorentz invariant account of the temporal development of the flashes, so this model is relativistically invariant only if “one limits oneself to considering possible entire distributions of flashes, renouncing an account of the coming into being of the actual distribution of the flashes” [25]. A theory with laws governing entire distributions of flashes, rather than the temporal coming-into-being of the flashes, would be temporally nonlocal in the atemporal sense, and hence it seems that a temporally nonlocal approach is more or less mandatory if one wishes to achieve a Lorentz invariant version of the GRW flash ontology. A similar dilemma arises in the context of causal set theory, which we will examine in detail in Section 6.2. We would conjecture that this point is true more generally: to achieve relativistic covariance in an interpretation of quantum mechanics, it will usually be the case that one has to abandon the notion of temporally local “coming-into-being.” (The Everett interpretation might be raised as a counterexample, but since the evolution postulated by the Everettian view takes place on configuration space rather than spacetime, it remains unclear what one should say about temporal locality and “coming-into-being” in that theory).

### 4.2. General Relativity

This point is even clearer in General Relativity (GR), where a solution to the Einstein field equations is not a state at a given time but rather an entire spacetime, a full history of a universe. It is tempting in view of this fact to argue that general relativity forces us to take an atemporal, temporally nonlocal viewpoint, but such a conclusion is not inevitable, because it has been shown that Einstein’s equations are *compatible* with a well-posed initial value problem. We can split the Einstein equations into a set of constraint equations (the equations for which both indices are spatial) and a set of evolution equations (the equations for which one index is temporal); then, given a smooth three-manifold Σ and a set of initial conditions on that manifold which satisfy the constraint equations, there exists a unique globally hyperbolic solution to Einstein’s equations—obtained by evolving the conditions on Σ forwards and/or backwards using the evolution equations—for which Σ is a Cauchy surface, meaning that the conditions on this surface determine the future and past uniquely [26,27,28]. This makes it possible to interpret general relativity as a temporally local theory with a kinematical state space restricted to the set of states which satisfy the constraint equations and a dynamics given by the evolution equations.

Of course, this reformulation will work in our actual universe only if the initial state of the actual universe does indeed satisfy the constraint equations. Do we know that this is the case? Arguably, yes—we know that on any hypersurface embedded in a spacetime which satisfies the Einstein equations, the conditions on the hypersurface must satisfy the constraint equations [26,27,28], so if we take it for granted that the universe as a whole satisfies the Einstein equations, then we can conclude that the initial state of the universe must satisfy the constraint equations. Nonetheless, something may be learned from considering the possibility of universes where the initial conditions do not satisfy the constraint equations. A key feature of the initial value formulation is that the constraint equations must be preserved under the evolution equations: if we vary the constraint equations while keeping the evolution equations the same, then in general we will find that initial states belonging the the kinematical state space will be taken to states outside the kinematical state space by dynamical evolution. This means that dynamics and kinematics are not fully independent in the initial value formulation of general relativity.

As Wharton points out [29], the traditional view of temporally local time evolution would have it that the laws of nature really do work like an initial value problem: the universe is presented with some initial state and must evolve it forward to produce a final state, just like a computer presented with an initial value and programmed to predict some value at some later time. The computer is not allowed to refuse the given value on the grounds that this is not the type of problem it prefers to deal with, and likewise, the dynamical laws of nature are not allowed to pick and choose the initial state on which they operate. Thus, even though general relativity can be given an initial value formulation, it is not at all clear that the traditional picture of temporally local time evolution can be maintained within this formulation, and thus general relativity may fit more naturally within a temporally nonlocal picture. (We pause here to note that general relativity is not the only theory in which a difficulty of this kind arises; the set of allowed kinematical states in Maxwell’s electrodynamics is also subject to constraint equations, about which, one presumes, similar arguments could be made. We do not regard this as a weakness of our argument; indeed, it may be regarded as a further piece of evidence in favour of the view that the laws of physics are in fact temporally nonlocal).

#### 4.2.1. Objection: The Independence of Dynamics and Kinematics

One might object to the argument given in Section 4.2 on the grounds that the independence of kinematics and dynamics on which the computational picture of the universe depends was never really realised even in pre-relativistic physics—after all, in any reasonable theory, the set of kinematical states must take a mathematical form such that the action of the dynamical laws is well-defined on every state in the set, so kinematics and dynamics can never be wholly independent. Moreover, the kinematics of a theory often makes ineliminable reference to dynamical quantities—witness the appearance of velocities in the characterisation of Newtonian phase space [30,31]. To which we say, first, so much the worse for the traditional view of time evolution! We will return to this theme in Section 6.1; however, we will also note here that the dependence in general relativity is of a more problematic kind. In Newtonian dynamics, a state belongs to the allowed kinematical set if and only if the action of the dynamical laws is well-defined on that state. This is a simple mathematical feature which can straightforwardly be regarded as a property of an individual state: it is, at least prima facie, a *temporally local* property. On the other hand, in general relativity a state belongs to the allowed kinematical set provided that it can be taken only into other members of the allowed kinematical set under allowed dynamical evolutions. How is this set defined? Can we simply say that the actual initial state of the world is chosen arbitrarily and the allowed kinematical set is then simply equal to the maximal set of states into which this state can be taken by allowed dynamical evolutions? This would restore the original picture in which the universe is presented with an initial state that it is not allowed to refuse. However, such a move would be a reasonable only if it is the case that a generic initial state will in this way give rise to an allowed kinematical set governed by a set of constraint equations which are not only comparably simple (by some appropriate measure of simplicity) to the actual constraint equations but which also can be unified with the actual dynamical equations in such a way as to produce a GR-like theory which is comparably simple (by some appropriate measure of simplicity) to the actual theory of GR; otherwise it would seem an implausible coincidence that we ended up with a universe governed by the simple, elegant laws of general relativity from an arbitrarily selected initial state. The argument thus hinges on a technical question whose answer is not presently known so for now we will content ourselves with noting that the equations of general relativity were derived in large part by appeal to the criterion of simplicity [32,33], and so it would seem quite surprising if there were a multitude of equally simple theories which would split into two parts to give the same dynamical equations but different constraint equations. If this move cannot be made, it seems as though the “initial” state must have been singled out on the basis that it would give rise to a particularly simple allowed kinematical set—which means that the choice of initial state actually depends on the state of the universe later in its evolution, so temporal nonlocality seems to be sneaking in through the back door.

#### 4.2.2. Objection: Modality

One might also object to this argument of Section 4.2 on the grounds that there is something fishy about the modal step. Indeed, the argument is superficially similar to a well-known argument due to Gödel, in which he argued that time cannot be absolute in general relativity because “the compatibility with the laws of nature of worlds in which there is no distinguished absolute time ... throws some light on the meaning of time also in the worlds in which an absolute time can be defined” [34]. This argument is regarded as problematic: in particular, the modal step has been challenged by Earman, who pointed out that it is not clear that “absoluteness”, must be an *essential* property of time, and therefore perhaps we should simply say that the status of time varies along with the distribution of matter, so time is absolute in worlds where there is a distinguished absolute time and not absolute in worlds where there can be no distinguished absolute time [35,36]. In the same way, one might object to our appealing to worlds with different constraint equations but the same dynamical equations on the grounds that perhaps the dynamical equation should be varied along with the allowed kinematical set in such a way to ensure a suitably simple GR-like theory. However, the difficulty with the modal step of Gödel’s argument stems precisely from the fact that in general relativity, spacetime and matter are not independent and therefore it is not reasonable to expect that questions about the nature of spacetime can be entirely divorced from facts about the constitution of matter in a particular universe; by contrast, on the traditional conception of time evolution, kinematics and dynamics are supposed to be independent, and therefore if this picture is correct it should be permissible to draw conclusions on the basis of holding the dynamics constant and varying kinematics, as we have done here.

### 4.3. Objection: Spacetimes That Are Not Globally Hyperbolic

Finally, one might worry that since we have only discussed the Cauchy problem in globally hyperbolic spacetimes, our argument might fail to go through if one allows the possibility of spacetimes that are not globally hyperbolic. We will not consider this case in detail here, but it seems likely that allowing spacetimes which are not globally hyperbolic would actually strengthen our argument. It is known that some but not all spacetimes with closed timelike curves admit a well-posed initial value problem, [37] yet a number of physicists have had the intuition that the laws of nature should not allow the existence of closed timelike curves, and to achieve this within the initial value formulation whilst not ruling out spacetimes which are not globally hyperbolic, it is necessary to place further constraints on the initial conditions to ensure that no closed timelike curves can be produced under the dynamical evolution [38]. Alternatively, one might want to allow closed timelike curves under the stipulation that they must be self-consistent, meaning that they do not produce “grandfather paradoxes” or comparable physical contradictions; and, again, this requires us to place constraints on the initial conditions to ensure that the local initial state can be extended to be part of a global solution which is well-defined throughout the non-singular regions of spacetime [38]. Either way, the specification of the allowed initial conditions once again makes reference to what are most naturally construed as global properties of an entire solution rather than temporally local properties of the initial state, which seems to support the temporally nonlocal viewpoint.

## 5. Three Options for Temporal Nonlocality

In this section we return to the three types of temporal nonlocality that we identified in Section 2.1, and review some relevant recent results.

### 5.1. Non-Markovian Laws

If the laws of nature do indeed prescribe a temporally local time evolution for the universe, this evolution must have the Markov property—that is, it must be possible to determine the probabilities for future evolution solely from the present state, without needing to know anything about the history [39]. However, we have good reason to be cautious about the Markov property in the context of quantum theories, because Montina has shown that any ontological Markovian theory of quantum mechanics requires a number of variables which grows exponentially with the physical size [40]. Montina concludes: “In order to avoid the exponential growth of the number of ontic variables, we have one possibility, to discard some hypotheses of the theorem. In our opinion, the Markovian property is the only one sacrificable”.

Indeed, on reflection, it is clear that there is something rather strange about regarding quantum mechanics as a Markov process. As a general rule, Markov processes lose information over time, because details of the system’s history which fail to be recorded are subsequently no longer accessible. However, the dynamics of unitary quantum mechanics is reversible, so if we take the quantum formalism literally, we conclude that in the absence of measurement, information about the past is never strictly lost—it just gets more and more spread out due to decoherence. Moreover much of this information will end up being stored in global properties of highly entangled systems which cannot be reduced to collections of properties of individual systems [41,42,43], so under the Markovian assumption we are forced to say that the information ends up stored in a “state” which is nonetheless not the state of any specific thing. However, if the formalism tells us that no information about the past is ever lost (except possibly in a measurement process) and also that most of this information usually cannot be attached to any single system or any particular physical location, then are we really saying anything particularly meaningful when we assert that the information is nonetheless all stored in the present state of the world? Under these circumstances, it is certainly more ontologically economically and arguably also more natural to say simply that measurement results at the present time depend *directly* on the history of the system, without any need for mediation via a nebulous state-like entity.

### 5.2. Retrocausality

Recently there has been renewed interest in “retrocausal” approaches to quantum theory [44,45,46,47,48], including a striking result due to Leifer and Pusey [7], expanding on an argument by Price [44], which demonstrates that if we insist on a certain kind of time-symmetry, quantum mechanics must allow for retrocausality, i.e., we must say that an experimenter’s decision to choose a certain measurement setting can influence the properties of particles in the past.

This increase in support for retrocausality is exciting in that it represents an attempt to move away from standard paradigms for the laws of nature. However, the invocation of retrocausality may also be a retrograde step, if the notion is employed as a way of salvaging temporal locality even in the face of increasing evidence against it. To see this, we must disambiguate several different ways of thinking about retrocausality. One important distinction is introduced in ref [47], where the author distinguishes between theories which are retrocausal only in the sense of invoking “reverse causality”, i.e., a simple global reversal of the direction of time, and retrocausal theories which allow causal influences from both the past and the future. But for our purposes, it is important to make a second distinction within this latter category, distinguishing between retrocausal theories which incorporate both backwards and forwards causal mediation, and retrocausal theories in which the universe is solved “all at once” without causal mediation in either direction. The first type of theory is perhaps best exemplified by the two-state vector interpretation of quantum mechanics [49], where measurement results at a given time still depend only on the state of the world at the time of the measurement, but this state now includes a “forward-evolving” state carrying information into the future from the past, and a “backward-evolving” state carrying information into the past from the future. This type of retrocausality, as in ref [45], still depends crucially on mediating states which carry information through time and thus such retrocausal theories look a lot like temporally local theories. However, retrocausal theories of the “all at once” type are naturally interpreted as temporally nonlocal, since although there is certainly a sense in which events in the future will have an influence on events in the past within such models, this influence need not be mediated by a record of those future events in the state of the world at the time of the measurement.

The distinction between these different ways of thinking about retrocausality is seldom made explicit in the literature on the subject, and thus it is not always straightforward to deduce into which camp various types of models are intended to fall. On the one hand, a number of recent models work by imposing global constraints and solving a constraint satisfaction problem across time [46,47], which tends to suggest the atemporal picture. On the other hand, it is common to motivate these models by arguing that retrocausality offers a means of salvaging *spatial* locality—the apparent nonlocality of the Bell experiments is to be explained by invoking a spatially local causal influence mediated via the *future* [44]—and this argument seems more at home within a picture in which influences from both the past and the future are mediated via a temporally and spatially local state. Similarly, the arguments of [7] are based on an assumption that Leifer and Pusey refer to as “λ-mediation”, which asserts that any correlations between a preparation and a measurement made on a system should be mediated by the ontic state of the system—by which, presumably, they mean the ontic state immediately prior to the measurement. The term “ontic state” is deliberately used in a general way here so as to make the result applicable to a wide range of theories, in much the same way as we refrained from specifying in detail what constitutes “the state of the world at time *t*” in our definition of temporal locality, but it seems clear that any reasonable precisification of these two notions would imply that the state of the world at time *t* includes, at the least, all the ontic states of all systems which exist at time *t* (if and when such ontic states exist), and therefore “λ-mediation” is essentially identical to the assumption of temporal locality. Leifer and Pusey acknowledge that their mathematical formulation of λ-mediation cannot be precisely correct for a theory which includes retrocausality, but they hope to replace it by something salvaging the notion that measurement results depend only on the present ontic state, which they regard as “a core feature of a realist theory”. Thus even within the retrocausality community the intuitive picture of mediation via a temporally local ontic state appears to persist.

However, this type of temporally local retrocausality requires a very finely tuned balancing act, because the backwards-evolving state must contain instructions which are compatible with the instructions from the forwards-evolving state—for example, the backwards-evolving state cannot enforce that a given particle must have some particular property if the forwards-evolving state enforces that it must have some mutually exclusive property. Formally, this balance is maintained because the future events which determine the backwards-evolving state are themselves determined by the forwards-evolving state at that time, but once we acknowledge this we are implicitly moving away from the picture of states evolving in fixed temporal directions and towards a kind of global coordination across time. In this picture, the assumption of temporal locality begins to seem highly artificial, and talk of a “backwards-evolving state” [49] or “influences that travel back in time” [7] look like rhetorical devices designed to preserve the appearance of temporal locality in a theory whose underlying structure is really temporally nonlocal.

We therefore suggest that the arguments of [7], along with other arguments that have been put forward in favour of retrocausality in quantum mechanics [44,46], are best interepreted as pointing us towards the atemporal type of temporal locality. Of course, this may be what has been intended by the proponents of retrocausality all along; if so, it would likely work in their favour to make this position clear. Indeed, it seems plausible that one major contribution to the reluctance of the wider physics community to take retrocausal theories seriously results from an implicit awareness of the tension that arises from attempting to balance information contained in forward and backward evolving states, and thus a significant conceptual barrier would be removed by moving explicitly to temporally nonlocal retrocausality.

### 5.3. Atemporal Laws

#### 5.3.1. The Lagrangian Schema

The paradigm of temporal locality is closely linked to what Smolin has dubbed the “Newtonian schema” [50], which is the assumption that “the universe is a computational mechanism that takes some initial state as an input and generates future states as an output” [29]. Wharton points out that even within classical physics, an alternative approach was available to us in the form of the “Lagrangian schema”, in which an experimental situation is described by a *Lagrangian*, a scalar function of various local parameters and their local derivative; the value of the Lagrangian for a given history is referred to as its “action”, and the classical action principle (an example of a “variational principle”) requires us to obtain predictions by choosing a set of boundary conditions and then extremizing the action [51]. For example, when the experimental situation under consideration is a ray of light travelling some unknown path, the Lagrangian is equal to the time taken to travel a given path, the boundary conditions are the initial and final positions of the ray of light, and thus we obtain Fermat’s principle, which states that light will always take the path which minimizes the total time of travel [51]. The two pictures can be related to one another via the Euler–Lagrange equations, which allow us to obtain a set of dynamical equations of the Newtonian type from any Lagrangian.

Furthermore, there exists a Lagrangian-schema formulation of quantum physics—namely, the path integral approach to quantum field theory, which generalizes the classical action principle by requiring us to calculate the probability of an event as a sum over contributions from all possible histories including that event; the contribution of a history is proportional to $e^{iS/\hbar }$, where *S* is the action for the history, equal to the time integral of the Lagrangian along the history. Like classical Lagrangian methods, the path integral formalism is a powerful calculational tool, and indeed, certain interpretative approaches advocate treating path integrals as the fundamental object of the theory [52,53].

Since the action is a property of an entire history rather than a feature of moment-by-moment temporal evolution, a naive interpretation of these Lagrangian-schema versions of our physical theories leads naturally to a temporally nonlocal view, and in particular, to the atemporal variant of temporal nonlocality. Of course, we cannot argue that the mere existence of the Lagrangian schema forces us to adopt this atemporal view, as for simple mechanical systems, the extremization of the action is both necessary and sufficient for the satisfaction of the Euler–Lagrange equations, and hence for such systems the two pictures are exactly equivalent. Even in more complex systems, the extremization is still always *sufficient* for the satisfaction of the Euler–Lagrange equations [54,55], so we can always pass from the Lagrangian schema to the Newtonian schema. However, let us observe that it is by no means inevitable that world should have been governed by laws of nature that admit these two formulations: a set of dynamical equations can be derived as the Euler–Lagrange equations of a variational principle if and only if, after transformation into a certain canonical form, the right-hand sides of all the equations are derivable by differentiation from a single function *H* [54,56,57]. If nature is really best described by something like the Newtonian schema, the fact that the actual laws of nature are indeed so derivable is simply an inexplicable coincidence, whereas this property can easily be explained if we postulate that the Lagrangian picture is in fact closer to reality and therefore all dynamical laws are necessarily the Euler–Lagrange equations of some variational principle.

A more formal version of this argument must wait upon an answer to the complementary question about the necessary and sufficient conditions under which “atemporal” laws of nature admit a formulation in terms of temporally directed dynamical laws; we hope to address this technical question in future work, but at present we must acknowledge the possibility that the answer will make the Lagrangian schema seem equally in need of explanation, in which case the comparison would yield no clear argument in favour of either approach. Nonetheless, it is sufficient for our purposes here to note that there exists a well-developed atemporal alternative to the Newtonian schema. Historically, the Lagrangian schema has been regarded as a mere mathematical tool, whereas the Newtonian picture of states evolving forward in time is treated as an approximate description of reality; however, there does not seem to be an obvious justification for this preference, other than a preexisting prejudice in favour of temporal locality. If we look past this prejudice, there seem to be good reasons to consider taking the Lagrangian schema seriously as a possible description of reality, and if we do so, we must necessarily take atemporal variants of temporal nonlocality seriously as well.

#### 5.3.2. New Models

The existence of the Lagrangian-schema formulations of both classical physics and quantum field theory makes it reasonable to argue that our best physical theories, as they currently stand, might be interpreted in a temporally nonlocal way. However, we can go further. Historically, the development of our physical theories has been constrained by the fact that research was by and large conducted within the Newtonian schema, and there has always been an expectation that new theories can be parsed in this framework—witness the great importance that was placed in the early history of General Relativity on showing that it could be given an initial-value formulation [27]. Thus we may well find that new avenues open up if we move to working directly on atemporal theories and indeed allow ourselves to consider theories which may ultimately turn out not to be susceptible to a Newtonian-schema formulation at all. Indeed, as noted in Section 5.2, a number of such models are already under development [46,47,48]. These toy models demonstrate that atemporal models are capable of reproducing in a natural way a number of prima facie puzzling features of quantum theory, such as the close resemblance between quantum wavefunction collapse and Bayesian updating [48], and thus provide motivation for further research into models of this kind.

## 6. Dynamics and Kinematics

We earlier identified the distinction between dynamics and kinematics as an important contribution to the status of temporal locality in physics. In this section we review recent work on this subject and discuss some resulting insights for the status of temporal locality in modern physics.

### 6.1. Spekkens on Dynamics vs. Kinematics

Spekkens has singled out the distinction between kinematics and dynamics as a potentially problematic feature of our standard physical paradigms: in [58], he argues that when new experimental data appears to falsify our existing theory, we can always choose freely whether to respond by altering the kinematics or the dynamics, and he gives a number of illustrative examples. He thus concludes that the distinction between kinematics and dynamics is doing no explanatory work in our theories, and appeals to ontological parsimony to motivate his call for physicists to move past this particular paradigm.

While we concur that the kinematics/dynamics split is problematic, we note that care must be taken with this line of argument to avoid slipping into conventionalism about the whole of science. Spekkens asserts that his approach “does not force us to operationalism”, because he advocates only the rejection of distinctions which we can freely transform away without changing empirical predictions, and which are therefore not doing explanatory work. However, as Quine has shown, it can reasonably be argued that physical theories have empirical consequences only taken as a whole, and that consequently we always have freedom to choose which element of a theory to change in response to new empirical evidence, which would suggest that by Spekkens’ criterion no distinction in any scientific theory is doing explanatory work [59,60]. Thus Spekkens’ line of argument would seem to lead to the conclusion that we should simply give up on trying to formulate theories whose ontologies are endowed with nontrivial structure, a conclusion which realists about science will surely wish to avoid.

To do so, we must understand why Spekkens’ argument has particular relevance in the context of the kinematical-dynamical distinction. In particular, let us reinforce that the distinction between kinematics and dynamics is not simply an individual element of some specific theory; the fact that it has become de rigueur to present new theories in this framework has made the kinematical-dynamical split into a meta-principle which physicists educated in this tradition may well regard as a defining feature of any meaningful scientific theory. By pointing out that the kinematical-dynamical distinction is not forced on us by any empirical evidence, Spekkens demotes it from a meta-principle back to a specific ontological hypothesis which should be subject to the same scrutiny and criticism as any other ontological hypothesis. The argument can then be understood as follows: as realists we choose to attach credence to certain ontologies, despite underdetermination by the empirical evidence, on the grounds of theoretical virtues like simplicity and explanatory power, and the same sorts of assessments should be applied to the distinction between kinematics and dynamics. Since theoretical distinctions in general do not have empirical content in and of themselves, it is no good insisting on a distinction between kinematics and dynamics in advance of specifying a particular ontology: we must evaluate the theoretical virtues of complete ontologies, some of which may incorporate such a split, others of which may not.

For clarity, at this point, we must mention a different way of thinking about the kinematics/dynamics distinction that has arisen through recent work on the philosophy of special relativity. In this tradition, “what it means for a phenomenon to be kinematical ... is that it is nothing but a specific instance of some generic feature of the world ... (and that) there is nothing more to learn from that particular phenomenon, neither about the specific system in which it occurs nor about the generic feature it instantiates”. In other words, the dynamics/kinematics distinction is regarded as a stipulation about which things need to be explained and which things can be taken for granted. This is clearly quite a different concept from the notion of kinematics and dynamics that we have thus far referred to in this article, and to which Spekkens’ argument pertains. Certainly it presupposes much less—in particular, such a distinction would still be perfectly meaningful within a theory which does not postulate a space of states and a set of evolutionary laws, whereas the more traditional way of distinguishing between kinematics and dynamics would be inapplicable in such a case. Nonetheless, we conjecture that similar arguments can be made about this more general distinction. Brown points out that “the distinction between kinematics and dynamics is not fundamental” and cites Pauli as making the same point in 1921, and, again, once this point is accepted it seems unreasonable to demand that all new theories must be presented in the framework of kinematics and dynamics: we may well find it heuristically useful to employ such a distinction in any particular case, but the judgement of its usefulness must be made in context, not in advance of the specification of a theory. We leave a more detailed development of this line of argument to future work.

### 6.2. Example: Causal Set Theory

As an example of a theory in which the kinematics/dynamics distinction may be less useful, consider the case of causal set theory, an approach to quantum gravity which holds that spacetime is fundamentally discrete. The “state space” of this theory is the space of causal sets—that is, sets of spacetime events with a partial order event defined over them. A causal set is, essentially, an entire history of a universe, with time and space being emergent from the partial order between pointlike events. However, it is not sufficient for the theory to simply specify this kinematical state space, because without further restrictions we will find that the majority of causal sets do not give rise to any low-dimensional emergent spacetime (a spacetime is said to emerge from a causal set iff it *faithfully approximates* the causal set—that is, we can embed the causal set into the spacetime in such a way that the causal relations are preserved (*x* lies before *y* in the partial order iff the embedding of *x* is in the past lightcone of the embedding of *y*), and on average one element of the causal set is mapped onto each Planck-sized volume of the spacetime, and the spacetime does not have structure at scales below the mean spacing of the events) so we must add in some way of singling out the permissible causal sets.

The standard way of doing this is to impose “dynamics”, such as the classical sequential growth model in which elements are probabilistically added to the set one by one [61]. Proponents of causal set theory like to advertise it as an advantage of their approach that this dynamics provides us with a relativistically covariant notion of “becoming”, allowing us to rescue the notion of temporal becoming and hence salvage our intuitive notion of time [62]. However, this claim cannot quite be taken at face value, because we encounter a difficulty akin to Smart’s objection to the A-theory of time. Smart famously pointed out that if time really passes, we ought to be able to specify the rate at which it passes, which would require a second time-dimension with respect to which the passage of ordinary time can be measured [63]; and likewise in causal set theory, talking about the growth of the causal set seems to presuppose an external time dimension in which this growth can take place. The difficulty is all the more pertinent since the founding principle of the theory is that spacetime supervenes on the causal set [64] and thus proposing a dimension of time external to the causal set would seem to undermine the whole project. Rideout and Sorkin attempt to get around this by arguing that the birthing of events should be regarded as “constituting” time rather than occurring in time [61], but this seems like overkill: since spacetime supervenes on the causal set, a complete causal set already “constitutes” time, without any need to add in a process of growth. Furthermore, to ensure that the growth process satisfies general covariance, it is necessary to impose the requirement of discrete general covariance on the dyamics, meaning that the probability of reaching a particular final causet is independent of the path taken to reach that final causet—i.e., the probability does not depend on the order in which the elemets of the causet were “birthed”. It is standard to interpret this by saying that there is no fact of the matter about which path was taken—the choice of path is pure gauge [61]—but this makes it implausible to regard the growth of the causal set as a real physical process, since probabilities are ultimately attached to the causal sets themselves rather than to the transitions that occur during the supposed growth [65,66]. Wütrick and Callender argue that these considerations simply show that modern physics requires us to adopt a ‘novel and exotic’ notion of becoming in which we are generally prohibited from saying which elements of the causal set exist at any stage of its growth [66]. However, this novel notion of “becoming” is so far removed from our intuitive notion of becoming that it is doubtful whether it can really be said to salvage our intuitive notion of time; moreoever, given that the dynamics cannot be taken literally, there is prima facie no way in which the growth of the causal set could even serve to explain why we have the subjective experience of becoming. The growth model, in fact, does not seem to add anything to the theory in terms of explanatory power: insofar as the dyamics succeeds in explaining why certain causal sets are permissible while others are not, and/or why the world is constituted by one causal set rather than another, the real explanatory work is done by the final probability distribution over causal sets rather than by the process of growth.

Thus it seems that all the growth picture is really doing is making causal set theory subjectively more palatable by soothing our uneasiness about attaching probabilities to entire courses of history, and, of course, allowing the causal set theorists to express their theory in the traditional framework of ‘kinematics vs. dynamics”. Thus, although it is *possible* to make a distinction between kinematics and dynamics within causal set theory, in this particular case the distinction seems not to be very useful and may in fact be holding us back from understanding the theory properly: perhaps the causal set theorists would do better to embrace the global nature of their theory and explicitly attach probabilities to entire causal sets, retaining the “growth” dynamics only as a calculational tool or perhaps even getting rid of it entirely in favour of a different way of calculating the relevant probabilties.

We have singled out the causal set approach here because the awkwardness of attempting to distinguish between kinematics and dynamics is particularly clear in this context, but we would contend that similar points apply more generally. Theories should not be forced into the kinematics-dynamics framework if they are not a natural fit for that framework: this practice imposes an artifical form of temporal locality on theories which are not inherently temporally local in their mathematical structure, which is likely to impede both understanding and also further theoretical progress.

## 7. Temporal Bell Inequalities and Entanglement in Time

The stark differences between contemporary attitudes to spatial and temporal locality can largely be traced back to the existence of Bell’s inequalities and the fact that quantum mechanics is known to violate them [67], an experimentally verified fact which has led the physics community to at take seriously the possibility that spatially nonlocal processes may exist. Of course, the implication is not undisputed; although a number of experimental loopholes in Bell’s theorem have been closed in recent years [68,69,70], there remain untested assumptions, such as the possibility that the choices of measurement on the two sides of the apparatus are not truly independent [71]. Furthermore, proponents of the Everett interpretation claim their approach can account for the Bell statistics in a spatially local way, and antirealists can avoid spatial nonlocality simply by denying that there exists any underlying process, local or otherwise, which accounts for the measurement statistics. However, each of these ways around the conclusion of the theorem requires us to accept a fairly extreme proposition of one type or another, so it is fair to say that, conditional on a set of assumptions which seem very plausible to many people, the violation of Bell’s inequality does indeed imply the existence of spatial nonlocality.

Thus it is very natural to consider whether some analogous set of equations are violated in the temporal case. The first point to be made is that the derivation of Bell’s theorem assumes both spatial and temporal locality. If we relax the assumption of temporal locality, then we could say, for example, that the result of the measurement may depend directly on the state of the system being measured at times other than the time of measurement, including *future* times: as we noted above, the proponents of retrocausality have used this possibility to explain the violation of the Clauser–Horne–Shimony–Holt (CHSH) inequality via a local interaction which is mediated via the future [44]. Thus, it is not really fair to say that we have better evidence for spatial nonlocality than temporal nonlocality: we have exactly the same evidence for both. However, physicists have largely chosen to respond to this evidence by discarding spatial locality and retaining temporal locality (or indeed by arguing that we can salvage both), and therefore to have convincing evidence that points specifically to temporal locality, we would need not just a temporal analogue of Bell’s inequalities, but a stronger result which shows that spatial nonlocality is not enough to explain the empirical results of quantum mechanics: the quantum world must be temporally nonlocal as well.

There exist several inequalities—mostly governing sequences of measurements performed on a single quantum system—which have been referred to as “temporal Bell’s inequalities”, and we will now consider whether any of them might be capable of providing the right sort of evidence. First, it should be clear that in the context of repeated measurements on a single quantum system, the assumption of temporal locality alone will not allow us to derive anything, since we can always choose to retain temporal locality by assuming that the entire history of a system is recorded in its present state. Thus, to obtain meaningful results, some further assumption *p* must be made, so we are never going to obtain a result stronger than “if this inequality is violated, then either ¬p, or quantum mechanics is not temporally local”. For this to provide a convincing argument in favour of temporal locality, *p* would need to be an assumption so plausible that many people would be willing to abandon temporal locality before abandoning *p*.

In the case of the Leggett–Garg inequalites, the additional assumption is “macrorealism”, which is the claim that a macroscopic object is at any given time in a definite ontic state and it is possible to determine which state it is in without changing the state or the subsequent system dynamics. Macrorealism is a strong assumption—too strong, in fact, for our purposes, because the only measurements referenced in the Leggett–Garg inequalities are measurements which reveal the definite ontic state of the system at the time of the measurement. As noted in Section 5.2, it is reasonable to assume that the state of the world at time *t* includes all the definite ontic states of all systems which exist at time *t*, and thus by definition the measurements referenced in the Leggett–Garg inequalities are only allowed to depend on the state of the world at the time of the measurement, which makes temporal locality irrelevant: whether or not the world is temporally nonlocal in general, for this specific type of measurement there is no freedom for the measurement result to depend on anything other than the present state of the world. There exist later reformulations of the Leggett–Garg inequalities which replace macrorealism with a weaker assumption, but most of these reformulations retain the assumption of “operational eigenstate realism”, that is, the assumption that quantum systems are necessarily in states in which the quantity being measured has a definite value which is revealed deterministically by the measurement, and again this makes the assumption of temporal nonlocality irrelevant [72]. A similar issue arises for the set of temporal Bell inequalities derived in [73], and used to demonstrate the phenomenon of “entanglement in time”. Here the derivation depends on temporal locality and also “realism”, defined as the assumption that measurement results are determined by hidden properties that the particles carry prior to and independently of observation; one assumes that the state of the world at the time of the measurement would be expected to includ these “hidden properties” and, thus under this assumption measurement results can depend only on the state of the world at the time of the measurement, so the auxiliary assumption already implies temporal locality, in the sense in which we have used the term.

Thus, if we are to derive a temporal Bell’s inequality has something to say about temporal locality in particular, we should look for an assumption *p* which does not itself imply temporal locality. A possible candidate is put forward in [74]: here, the derivation of the inequalities is based on the assumption that the results of measurements on a system of dimension *d* should be simulable by an ordered set of classical systems with no more than $\log_{2}\left(d\right)$ bits of communication between any consecutive pair of systems. It is helpful to split this assumption into two parts: first, the total amount of information about its history that can be carried forward in time by a quantum state of fixed dimension is upper bounded by $\log_{2}\left(d\right)$ bits; and second, the result of a measurement on the system is statistically independent of all information about its history which is not stored in its present state, i.e., the measurements in question are temporally local. Ref. [74] use this assumption to derive a bound on the minimum dimension of a system which can solve a certain sort of sequential problem, and then show that the problem can be solved by quantum systems of dimension smaller than this bound, indicating that quantum mechanics does not satisfy their assumption. This is exactly the kind of result needed to provide an argument for temporal locality: if we find it sufficiently unpalatable to postulate that quantum states may carry information greater than $\log_{2}\left(d\right)$ forward in time, we will have to conjecture instead that the later measurement results depend directly on earlier measurement settings and outcomes without being mediated via information carried forward in the state, and thus we may regard the violation of this inequality as a direct demonstration of temporal nonlocality at work in quantum mechanics, in the same way that a Bell experiment is a direct demonstration of spatial nonlocality at work in quantum mechanics. Admittedly, it may not be the case that there are many people who find the bound $\log_{2}\left(d\right)$ more intuitively plausible than temporal locality, but at least the result seems to be of the right form.

### The Problem of Records

It may seem that the existence of records of past measurement results must always stymie any attempt to use the violation of some inequality to prove that the world must be both spatially and temporally nonlocal, since even if we do make an assumption like that of [74] to the effect that a given system can only carry a bounded amount of information forward in time, a proponent of temporal locality could always claim that a given result depends on the record of a given measurement result stored *elsewhere* in the present state of the world, rather than directly on the past events constituting the measurement. After all, in practice such records are very difficult (perhaps impossible!) to erase, and in any case, if a past measurement result could be permanently erased so that no record of it existed in the state of the world at the time of the next measurement, then we would never be able to observe the violation of the relevant inequality, since we could never have all the necessary results available to be compared at the same time.

We suggest the best way of resolving this difficulty is to adopt a halfway position inspired by our discussion in subsection “The Pragmatic Argument”. The problem that we are facing can be understood as a particular instance of the general problem identified by Einstein: if spatial locality is simply abandoned wholesale, it becomes impossible to identify and control all the factors which might possibly influence the results of an experiment, and thus we lose the ability to draw meaningful conclusions from experimental results. Therefore, as noted in subsection “The Pragmatic Argument”, to make progress we must assume that there are limits on spatial nonlocality. The most straightforward approach is to assume that the world is only as spatially nonlocal as quantum mechanics says it is, because then, provided the system being measured is in a sufficiently pure state, we can justify the assumption that the result of the measurement is independent both of records stored elsewhere in the world and of the state of the observer’s brain. The resulting inequality will still be theory dependent, but at the very least the violation of such an inequality, assuming we are not willing to abandon the assumption that quantum states of dimension *d* may only carry $\log_{2}\left(d\right)$ bits of information forward in time, would force us to say either that the world must be temporally nonlocal or it must be more spatially nonlocal than quantum mechanics currently suggests.

An alternative would be to assume that we need only worry about spatial and/or temporal entanglement when the systems concerned can be connected via some reasonably simple spatiotemporal path, as for example in the case of two entangled particles which have interacted locally at some point in their shared past. This would have the advantage of removing any dependence on the present formalism of quantum mechanics, which may be desirable given that we do not know how much of that formalism would survive the move to a temporally nonlocal context, but on the other hand to make the criterion precise we would likely need a reasonably concrete proposal for an alternative theory, and at present only toy models are available to us for this purpose.

## 8. Conclusions

There already exists a small body of interesting work examining the possibility of what might be interpreted as temporally nonlocal approaches to quantum theory, although most of it has not yet reached the mainstream. Wharton, advocating the view that “the universe (runs) not as a computer, but as a global four-dimensional problem that (is) solved all at once” [29], has made progress with retrocausal models [75,76,77]; the consistent histories approach offers an approach to formulating laws of nature which constrain entire histories rather than moment-by-moment evolution [78,79], although there are a number of significant conceptual difficulties to be resolved, not least the question of what the probabilities prescribed by the theory are probabilities for [80]; and Ref. [81] puts forward a theoretical model, in which “*one particle at N times is ... equivalent to N (entangled) particles at one time*”, which, by emphasizing the parallel between spatial nonlocality and time-evolution, seems to lead naturally to a temporally nonlocal view. Similarly, there exist interpretations of quantum mechanics whose ontology consists entirely of pointlike events, such as the GRW flash ontology [23,24] or Kent’s solution to the Lorentzian quantum reality problem [82], and one possible interpretation of these approaches would be to say that they have done away with the need for an ontic state as the carrier of information from the past to the future and hence should be regarded as temporally nonlocal.

This existing work is very promising, but we would argue that it does not go far enough. These approaches have been postulated as part of the project of interpreting the existing framework of quantum mechanics (and/or quantum field theory), and yet, once we accept that the universe may be generically nonlocal across both time and space, it becomes at least plausible that quantum theory as we know it is simply the local limit of a global theory which applies constraints across the whole of space and time. This means there is scope to be more ambitious: temporal nonlocality may ultimately point us not just to a new interpretation of quantum mechanics but to a new theory altogether.
